# A Framework to Guide Defining an Upper Threshold of Crystalline Vitamin B12 in Foods and Food Supplements

**DOI:** 10.1007/s13668-025-00622-7

**Published:** 2025-02-13

**Authors:** Rima Obeid, Juergen Geisel, Klaus Pietrzik, Emmanuel Andres

**Affiliations:** 1 Department of Clinical Chemistry and Laboratory Medicine, Saarland University Hospital, 66424 Homburg, Germany; 2 Department of Nutrition and Food Science, Rheinische Friedrich-Wilhelms University, Endenicher Allee 19B, 53115 Bonn, Germany; 3 Department of Internal Medicine, Diabetes and Metabolic Diseases, Hôpitaux Universitaires de Strasbourg, Strasbourg, France

**Keywords:** Intake, Drug, Food supplement, Pharmacological effect, Safety, Vitamin B12

## Abstract

**Purpose of review:**

To define an intake threshold of vitamin B12 from food supplements that is sufficient to maintain normal body functions, but it does not cause pharmacological effects.

**Recent findings:**

We used studies on the amount of B12 absorbed following oral B12 application and non-comparative case-series studies to synthesize evidence on pharmacological effects of oral B12 (between < 10 µg and 3000 µg) in people with manifested deficiency. There is a dose-dependent intestinal absorption of B12 and in the same time effects on body metabolism and functions. Food supplements providing ≤ 20 µg B12 daily are unlikely to cause pharmacological effects, while 50 µg might correct abnormal biochemical markers in some deficient patients. Foods for special medical purposes for people who cannot absorb B12 may contain 100 µg to 150 µg B12. This dose may ensure 1–4 µg of the vitamin reaching the circulation on a daily basis independent of intrinsic factor. Dosages ≥ 200 µg/d should be considered as drugs that can correct anemia, metabolic markers and clinical symptoms.

**Summary:**

The content of vitamin B12 in food supplements should not exceed 20 µg. In addition, people with deficiency should receive appropriate medical treatment with high dose B12.

## Introduction

People who regularly consume food supplements may exceed the recommended dietary intake of some nutrients by several folds [1]. Food supplements underlie country-specific regulations but often a lack of oversight authorities responsible for post-market surveillance to ensure safety for the general population [2]. It has been therefore argued that the amount of key nutrients should be limited in food supplements [1].

Vitamin B12 (cobalamin) is a water-soluble B-vitamin with unique roles in metabolism and body functions. The recommended dietary intake for vitamin B12 in adults is 4.0 µg/d according to the European Food Safety Authority (EFSA) [3] and 2.5 µg/d according to the United States National Academy of Medicine (NAM) [4]. A western diet provides approximately 4–7 µg/d of vitamin B12, an amount that is considered sufficient for maintaining normal vitamin B12 status in healthy adults [5–7]. Vitamin B12 requirements increase by 0.5 to 1.0 µg/d during pregnancy and lactation compared to adult’s population [3]. There are currently no specific intake recommendations for subgroups such as elderly people and people with certain illnesses. A Tolerable Upper Intake Level (UL) for vitamin B12 has not been defined due to lack of appropriate data. Massive dosages of vitamin B12 (e.g., ≥ 250 times the recommended intake) are being placed on the market as food supplements or drugs (containing cyano-, methyl- or adenosyl-cobalamin).

The maximal amount of vitamin B12 that fits into the distinct product category of food supplements is not adequately defined [4, 8]. The German Federal Institute for Risk Assessment (BfR) suggested that food supplements should not provide more than 25 µg vitamin B12 per day, assuming that if people would consume 2 sources of vitamin B12 per day, they might achieve a total daily intake of 50 µg [1]. A maximum daily intake of 50 µg of vitamin B12 has not been linked to adverse effects in the population or subgroups of the population.

The aim of this review is to define a threshold of vitamin B12 intake that is sufficient to maintain normal body functions, but is not likely to cause pharmacological effects. Moreover, a least vitamin B12 intake level whose effect is discernible on clinical signs and symptoms of vitamin B12 deficiency will be defined based on historical data on people with manifested vitamin B12 deficiency. Finally, we aimed to define a maximal amount of B12 that should not be exceeded in single food supplement products. We approached this question by reviewing human studies on B12 absorption and data from non-comparative case-series studies on clinical effects of oral vitamin B12 in patients with manifested B12 deficiency (e.g., pernicious anemia as a classical disorder of vitamin B12 absorption). This paper does not aim to question the present recommendations of diagnosing and treating B12 deficiency, neither to define the “lowest effective dose to treat manifested B12 deficiency”.

## Food Supplements, Foods for Special Medical Purposes and Drugs: Regulatory Aspects

Food supplements are consumed on top of the regular diet. The term “supplement” is widely used, but it does not distinguish between food supplements and vitamins for therapeutic use. In many countries, food supplements are regulated as foods that are intended to “maintain an adequate intake of nutrients or to support specific physiological functions, but are not intended to replace the diet or to act as medicinal products”. Food supplements should not exert pharmacological, immunological or metabolic effects and they are not intended to modify physiological functions or to treat or prevent diseases.

In Europe, foods for special medical purposes (FSMP), also known as medical foods in the U.S., constitute an additional group of food supplements that are increasingly being characterized under food laws, rather than as drugs. FSMPs intend to feed patients who have medically determined nutrient requirements that cannot be met by modification of the normal diet alone or by using non-FSMP foods such as usual food supplements and fortified foods. FSMPs target people who have a well characterized disease or medical condition such as short bowel syndrome, renal disease or specific inherited metabolic disorders. According to the European legislation, FSMPs are intended for patients with a limited, impaired or disturbed capacity to take, digest, absorb, metabolize or excrete ordinary foods, or certain nutrients or metabolites; or for patients with medically-induced nutrient requirements whose dietary management cannot be achieved by modification of the normal diet alone [9]. FSMPs should be used only under medical supervision and must carry labelling information about their intended use [10].

Drugs are generally defined as substances or preparation of substances which are intended for use in or on the body and that have properties to cure, alleviate or prevent diseases or pathological conditions. Drugs can be administered to restore, correct or influence physiological body functions by pharmacological or immunological mechanisms. If a product has the ability to treat, cure, alleviate symptoms of a disease, or prevent diseases, it is considered a drug, even if it is “wrongly labeled” as a food supplement.

The categories of food supplements, FSMPs and drugs are mutually exclusive. However, therapeutic doses of vitamin B12 are currently marketed as food supplements, FSMPs or drugs [11].

## Vitamin B12 Physiology and Causes of Deficiency

Foods of animal origin are the main source of vitamin B12 for humans. The body stores are preserved by reabsorption of vitamin B12 excreted via the kidney and the bile. Vitamin B12 is needed for normal function of the hematopoietic system including normal production of blood cells such as erythrocytes and reticulocytes in the bone marrow. In addition, the vitamin is essential for the function of the central nervous system [12, 13]. In the cell, vitamin B12 is a cofactor for methionine synthase and methylmalonyl-CoA mutase.

Vitamin B12 deficiency causes elevated plasma concentrations of homocysteine and methylmalonic acid. Elevated concentrations of methylmalonic acid may correlate with the clinical symptoms, such as neuropathy [14], but may not be a good prognostic marker for the clinical picture [15]. Treatment with B12 corrects vitamin B12 markers, blood count, anemia and neurological symptoms. These pharmacological effects are caused by interaction of the vitamin with the cellular enzymes.

Low dietary intake of vitamin B12 such as in people who adhere to a vegan diet can cause vitamin B12 deficiency in the long term. In western populations, malabsorption disorders explain the majority of cases with vitamin B12 deficiency in adults and elderly people. Several gastrointestinal disorders such as pernicious anemia (IF antibodies), gastric atrophy, Crohn’s disease or bariatric surgeries can cause vitamin B12 malabsorption [16–20].

Interference with B12 absorption or metabolism may occur in people using medications such as metformin [21–27], proton pump inhibitors [28] or L-dopa [14]. Food cobalamin malabsorption may explain B12 deficiency in many elderly people (> 60 years) who cannot release vitamin B12 from foods [29, 30], while the IF-mediated absorption of small amounts of crystalline-B12 from fortified foods or food supplements can still be normal in those people.

## Current Practices of Diagnosing and Treating B12 Deficiency

Vitamin B12 deficiency can affect several organ systems such as the central nervous system, the peripheral nervous system, the digestive tract, and bone marrow. Macrocytic anemia is a typical hematological manifestation of B12 deficiency, although not expressed in all patients. Vitamin B12 deficiency causes disturbed DNA-synthesis and a delay in red blood cell maturation that becomes manifested as low red blood cell count and hemoglobin levels and elevated mean corpuscular volume. Other common manifestations of vitamin B12 deficiency are neuropathy, deficits of deep sensation, pain, and memory deficits. Also unspecific symptoms such as sore tongue, tiredness, mood disorders, and loss of appetite are observed. The variability of symptoms can cause a significant delay in diagnosing B12 deficiency. Concentrations of vitamin B12 markers such as methylmalonic acid, homocysteine, and/or vitamin B12 in blood are currently used to guide the diagnosis.

Subclinical or latent vitamin B12 deficiency is common in the general population and it needs to be diagnosed and appropriately treated. Therapeutic doses of B12 should start without any delay to prevent progression and precipitation of the neurological symptoms. The treatment with B12 can be personalized and it may last for a long time. People with vitamin B12 deficiency should be under regular medical supervision and the response to treatment should be monitored [31, 32].

## Why Should Food Supplements Not Contain High Dose Vitamin B12

Defining a maximal amount of vitamin B12 in foods and food supplements should balance between the benefits of successfully achieving the “nutritional needs” and the risks of overdosing or self-treatment in subgroups of the population [33]. Recently, the National Institute for Health and Care Excellence in the United Kingdom (NICE) raised concerns regarding using over-the-counter preparations containing vitamin B12 in people with vitamin B12 deficiency [31]. Uncontrolled use of vitamin B12 may slightly or temporarily increase circulating total B12 or active B12 concentrations without fully correcting the deficiency, thus causing the risk of under-treatment [31]. A self-medication can also mask the deficiency and delay the diagnosis of the underlying disorder and can cause some symptoms to become irreversible if the B12 dose is insufficient to fully correct the deficiency.

## Vitamin B12 Absorption 

### Absorption in Healthy People

Naturally occurring vitamin B12 is released from foods by chewing and the effect of saliva and digestive-enzymes. The stomach produces IF that captures vitamin B12 and facilitates its uptake to the enterocytes via the IF-receptor, cubilin. IF can bind between 3.0 µg and 6.0 µg vitamin B12 per meal [34] and may facilitate the uptake of approximately 1–2 µg of B12 to the circulation after each meal in healthy people. In people with normal liver stores (estimated to be 5000 µg B12), vitamin B12 that reaches the blood is used to maintain normal vitamin B12 status and B12-dependent physiological functions, such as the hematopoietic functions [35, 36]. An intake between 4 µg and 7 µg/d from natural foods or food supplements is sufficient for maintaining normal B12 status in healthy people [5–7].

**Fig. 1:**
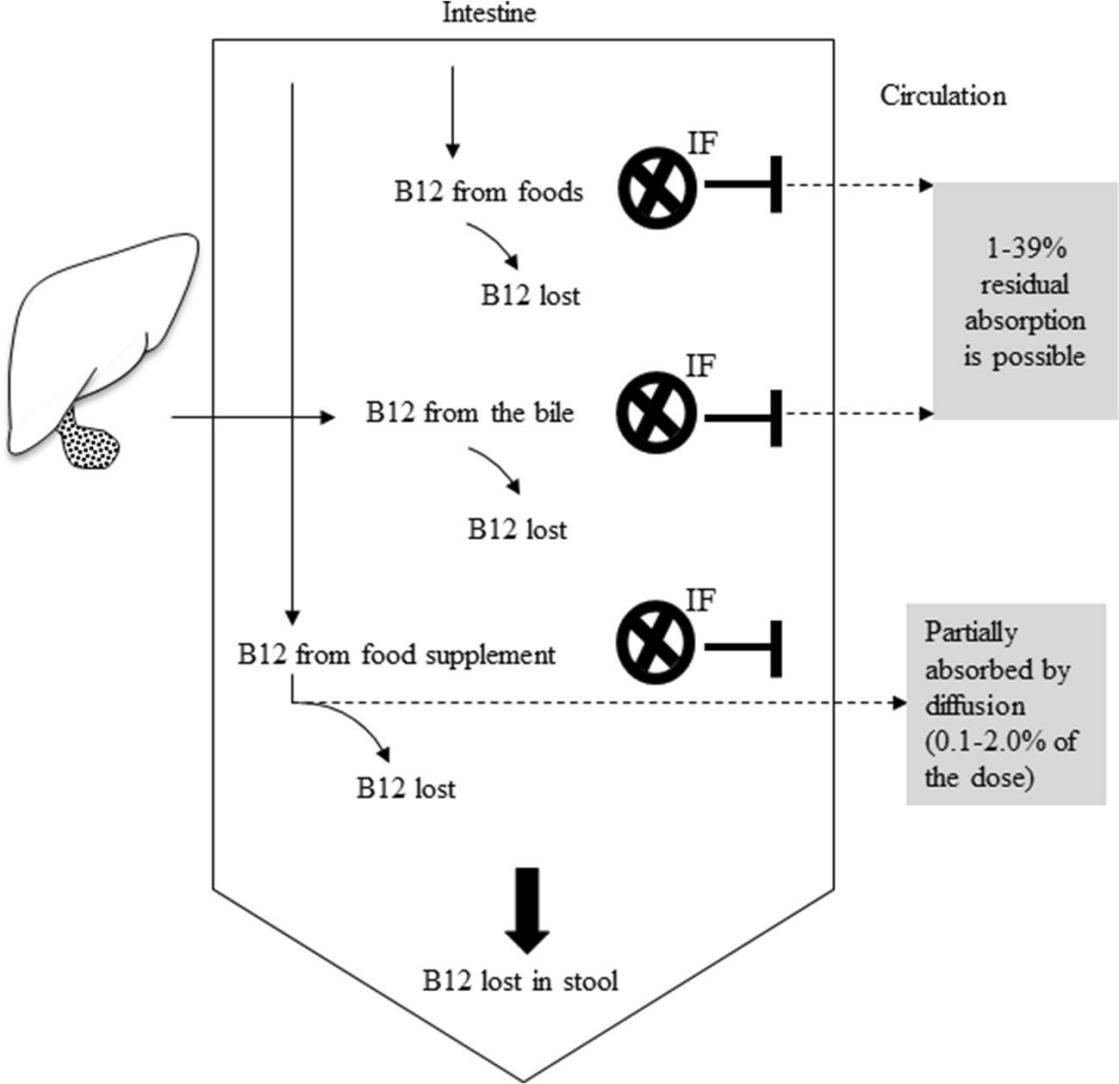
Vitamin B12 malabsorption (e.g., pernicious anemia) can cause B12 deficiency. Vitamin B12 from foods and food supplements cannot be fully absorbed due to lack of functional intrinsic factor. Vitamin B12 that is excreted into the bile is lost in stool instead of being reabsorbed. A small amount of vitamin B12 from food supplements (approximately 1% of the oral dose) can enter the circulation by simple diffusion

In people with normal B12 absorption, IF can mediate the absorption of vitamin B12 provided through food supplements. Different amounts of orally administered B12 (range 0.1%—2.0%) can pass from the gut to the circulation by simple diffusion. The efficiency of vitamin B12 uptake by passive diffusion depends on several factors such as the oral dose of B12 (higher absorption efficiency at lower doses) and duration and frequency of supplement use (more efficient absorption when split the dose). Passive diffusion contributes to B12 absorption when the amount of vitamin B12 in the pharmaceutical product exceeds the binding-capacity of IF and also when the IF-activity is absent (e.g., in people with pernicious anemia). In vitamin B12-deficient vegans and elderly people with food cobalamin malabsorption, the absorption of an oral dose of crystalline B12 is possible both via IF and passive diffusion (Fig. 1).

### Absorption in People with Pernicious Anemia

People with pernicious anemia are normally under medical treatment with high dose B12. In addition to the regular B12-treatment, they may use FSMPs as a dietary source of vitamin B12 on a daily basis.

B12-absorption can take place by an IF-mediated process and per diffusion (free B12 moves against the concentration gradient from the gut to the circulation) [37, 38]. Many patients with pernicious anemia may have some IF-binding activity that could explain between 1 and 39% residual absorption of B12 [37]. This could explain variations in the response to oral B12 therapy. In patients with pernicious anemia in remission, normal hematopoietic functions were maintained when doses between 1 µg/d and 5 µg/d of vitamin B12 were administered parentally (100% available in the circulation) [35, 36, 39]. Moreover, it has been reported that a single oral dose of 100 µg to 300 µg B12 [40–43] can cause between 1 µg and 3 µg of vitamin B12 to reach the circulation, despite between-individual variability in the fractional absorption (Table 1). Therefore, an oral B12 dose in the range between 100–300 µg/d appears to be sufficient for maintaining normal hematopoietic functions and liver B12 stores in people who cannot absorb the vitamin.

**Table 1:** Estimated vitamin B12 amounts absorbed from a single oral dose using different methods

In control subjects Mollin DL. 1959 [44] ^1^				
Single oral dose of labelled B12, µg		Number of subjects/number of observations	Range of B12 absorbed, µg	Mean of B12 absorbed in control subjects from the oral dose, µg
0.1		4	0.05–0.09	0.08
0.25		8	0.08–0.23	0.19
0.5		74/98	0.16–0.48	0.35
0.6		41	0.12–0.55	0.38
1		33	0.26–0.87	0.56
2		21/23	0.08–1.66	0.92
5		15/16	0.10–2.5	1.4
10		6/7	0.00–3.4	1.6
**In patients with pernicious anemia using radioactive B12 (Schilling test)**				
	Berlin H, 1962 [40]		Waife et al., 1963 [42]	Brody et al., 1959 [41]
B12 oral dose, µg	B12 absorbed, µg		B12 absorbed, µg	B12 absorbed, µg
1	0.075			
3	0.135			
10	0.39			
100	1.5			
150				2.00
200	3.3			
300			2.4	
400	5.4		4.8	
500	8.1		4.7	
800	8.7			
900			6.7	
1600	21			
3000	51			
5000	78			

^1^ Mollin DL. 1959 [44] tested the absorption of B12 in control subjects after a single oral dose of B12. The study used the fecal excretion method: The oral dose of radioactive B12 is given to the fasting subject, and the faecal excretion of radioactivity is estimated, the difference is the absorbed B12

## Pharmacological Effects Caused by Oral B12: A Dose–Response Relationship

Food supplements and FSMPs should not cause pharmacological effects. Whereas, treatment with oral vitamin B12 causes several pharmacological effects such as correction of hematological and metabolic abnormalities and/or clinical symptoms of deficiency [45–51]. The severity and cause of vitamin B12 deficiency influence the response to B12 dose. Patients with pernicious anemia in relapse (acute stage of deficiency) show generally a greater response to small amounts of the vitamin compared to patients in a remission phase. Early case-series studies with well documented response of hemalotogical symptoms and serum B12 to B12 treatment were reviewed to define a threshold of B12 intake that is not likely to cause pharmacological effects in people with manifested deficiency.

### Vitamin B12 Intake up to 50 µg/d

Using oral vitamin B12 dose between 3 µg/d and 6 µg/d did not prevent vitamin B12 deficiency in the majority of patients with malabsorption disorders [52–54]. Doses between 5 µg/d and 10 µg/d showed no detectable hematologic responses in deficient patients with pernicious anemia [55, 56]. While a dose between 5 µg/d and 17 µg/d caused a short-term hematological remission [57]. Suboptimal hematological response was also reported when 30 µg/d oral B12 was used [35]. A dose between 25 µg/d and 100 µg/d for up to 13 days caused a suboptimal induction of the reticulocytes [56].

Treatment with 50 µg/d oral vitamin B12 for up to 22 months in patients with pernicious anemia in relapse (n = 12) normalized hematological values within 2 months, but was not sufficient to maintain normal serum vitamin B12 values [58]. Another study reported significant clinical and hematological responses after ≤ 2 weeks of treatment with 50 µg/d oral B12 in 8 patients with pernicious anemia in relapse [59]. The reticulocytes response was delayed, prolonged and of a plateau type, while the bone marrow was generally megaloblastic in the majority of the patients treated with 50 µg/d oral B12 [59].

Therefore, a dose ≤ 20 µg/d can be used in food supplements because no (or no sustainable) pharmacological effects were seen in the majority of deficient patients who were treated with such a dose. A dose of 50 µg/d has shown minor hematological effects (stimulation of blood reticulocytes cells within 5–10 days and raise of serum vitamin B12) in some, but not all patients with pernicious anemia in relapse. Although, replenishing B12 stores may not follow a linear course when the B12-tissue stores are empty, if people with B12 deficiency (subtle or manifested), but without malabsorption disorders (such as in vegans or elderly with food cobalamin malabsorption) would use food supplements containing 50 µg/d B12, correction of signs and symptoms of B12 deficiency such as metabolic or hematological abnormalities may occur in the long term.

### Vitamin B12 Intakes > 50 µg/d and < 200 µg/d

Patients with pernicious anemia in relapse (n = 7) who used 100 µg/d for 15 days showed partial improvement of hematological markers and blood B12 concentrations [47]. In 1950, Meyer et al., argued that the minimal effective dose of vitamin B12 given orally to patients with pernicious anemia should be between 75 and 150 µg/d [60]. In a study among people with pernicious anemia in relapse who were initially treated with parenteral vitamin B12, patients who received 100 µg/d oral vitamin B12 maintained hemoglobin levels and red blood cell counts in the normal range during a follow up period of up to 2 years, suggesting that this dose could be suitable for FSMPs.

The effect of maintenance treatment with 100 µg/d vitamin B12 for 2 years on serum B12 concentrations was studied in 71 patients with pernicious anemia and compared to the effect of monthly i.m. injection of 100 µg B12 among 84 patients for the same follow up duration [61]. The results showed that 100 µg/d oral B12 was sufficient for maintaining normal serum B12 in patients with B12-malabsorption in remission [61]. Providing 100–150 µg/d oral B12 caused hematological remission in almost all patients, but some patients were unable to maintain their plasma vitamin B12 in the long term [41, 49, 62]. The reticulocytes number and hematocrit rise after 20 to 80 days of treatment with 150 µg/d oral B12 in patients with pernicious anemia in relapse [49]. Hematological effects that were seen after 10 days of starting 150 µg/d oral B12 were comparable to the effects seen after parenteral B12 [49]. In term of correcting serum concentrations of vitamin B12, treatment with 150 µg/d did not normalize serum B12 in all patients unless a daily oral dose of 1000 µg vitamin B12 was used [49]. Moreover, mild neurological relapse and glossitis were developed in one of 10 patients treated with 150 µg/d, before the 1000 µg/d oral B12 was started [49], suggesting that 150 µg/d is not sufficient to prevent neurological relapse in some patients.

Collectively, the studies discussed above clearly show that an oral B12 dose between 100 µg and 150 µg/d is likely to maintain normal hematology markers and normal serum B12 levels in the majority of people with malabsorption disorders in a remission phase, but may not prevent the deficiency and may not cause persistent pharmacological effects in patients who are in relapse. Therefore, a B12 dose between 100 µg and 150 µg may be considered suitable for the product category, FSMPs.

### Vitamin B12 Intakes of 200 µg/d and Higher

**Fig. 2:**
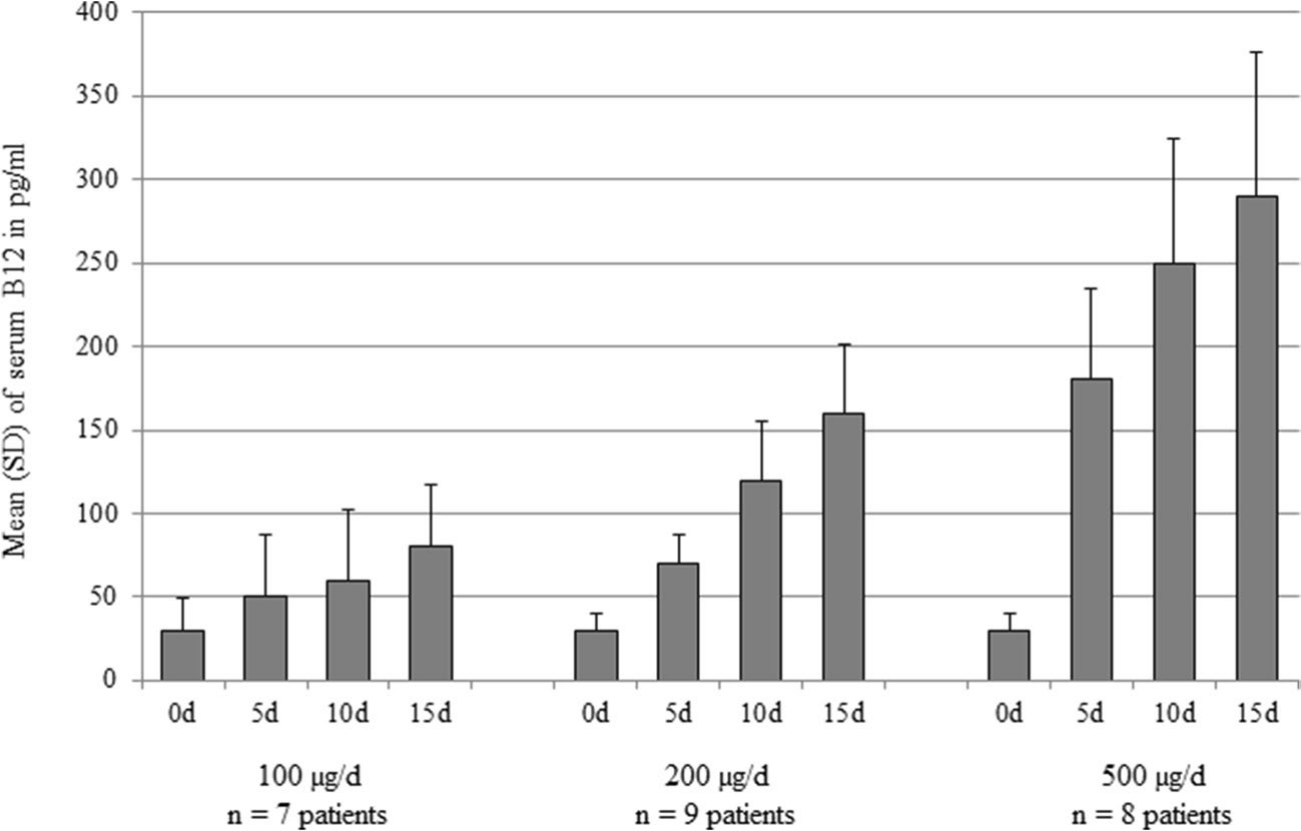
Effect of treatment with oral vitamin B12 (100, 200 or 500 µg/d) for 15 days on serum concentrations of vitamin B12 in people with B12 deficiency due to pernicious anemia [47]

In a study among patients with pernicious anemia in relapse (n = 17), oral B12 (100 µg/d, 200 µg/d, or 500 µg/d) was used without IF [47]. All patients showed a correction of serum vitamin B12 concentrations and the hematological parameters (hemoglobin, RBC counts and mean corpuscular volume) within 15 days treatment with any of the three dosages [47]. There was a dose-dependent increase in serum vitamin B12 concentrations within 15 days (serum B12 increased by + 80 pg/ml in the 100 µg/d dose; + 160 pg/ml in 200 µg/d group; and + 290 pg/ml in 500 µg/d group) [47] (Fig. 2). Continuing the treatment in 10 patients has shown that 200 µg/d and 500 µg/d were able to maintain hematological values and serum vitamin B12 for 3 to 13 months [47]. The effects of 500 µg/d followed by 200 µg/d (on serum vitamin B12) were more pronounced than the effect of 100 µg/d [47] (Fig. 2). The authors argued that a dose of ≥ 200 µg B12 may ensure achieving normal serum B12 in patients with malabsorption in relapse [47].

Normal hematological values were found among all patients with pernicious anemia in relapse who were treated with 100 µg/d, 200 µg/d or 500 µg/d oral B12 for up to 26 months [59]. None of the patients developed neurological complications during the observation time [59]. In patients who were treated with 500 µg/d oral B12 for 8 months then shifted to parenteral B12 (100 µg every 2 weeks) for additional 6 months, serum concentrations of vitamin B12 were similar under parenteral and oral treatments [59].

Another study has shown that 500 µg/d of oral B12 (compared to no B12-treatment) was effective in preventing B12 deficiency after 3 years of bariatric surgery [18]. Moreover, 500 μg/d oral B12 improved vitamin B12 deficiency-anemia in patients with total gastrectomy due to gastric cancer [48]. The same dose raised serum B12 within 3 months in approximately 92% of patients with gastrectomy who were B12 deficient [63]. Although 500 µg/d oral B12 is not considered sufficient for treating manifested B12 deficiency according to latest recommendations, it shows clear pharmacological effects and should be considered inappropriate for foods and food supplements including FSMPs.

Treatment with 1000 µg/d or 1500 µg/d in patients with total gastrectomy (e.g., after gastric cancer or gastric bypass surgery) was more likely to be associated with improved serum B12 compared to doses between 350 µg/d and 500 µg/d [63–66]. An oral dose of 1000 µg/d cyanocobalamin can improve neurological and hematologic symptoms and normalize vitamin B12 status markers (serum B12, homocysteine and methylmalonic acid) in deficient people [67–75].

A recent systematic review and meta-analysis showed a dose–response relationship between the oral dose of vitamin B12 (from 200 µg/d to 1000 µg/d) and the response of serum vitamin B12 and methylmalonic acid concentrations [46]. Andres et al., have shown that 1000 µg/d oral B12 normalized macrocytosis and serum B12 in elderly people with B12 deficiency within one month [73]. A recent observational study of 22 patients with pernicious anemia who were treated with 1000 µg/d oral B12 for 12 months has shown that serum B12 and methylmalonic acid were normalized within 1 month [76]. Many patients showed hematological and/or neurological improvements starting from 3 months after initiating the treatment [76].

Taken together, high dose B12 (e.g., 1000 µg) are inappropriate for food supplements or FSMPs because they cause marked metabolic and clinical improvements in deficient patients. Treatment of patients with manifested vitamin B12 deficiency with oral and parenteral vitamin B12 has been intensively discussed in the literature [31, 32] and is not the subject of the present article.

## Limitations of the Methods and Evidence

The present review aimed to define an oral B12 intake that is sufficient to maintain physiological body functions, but is not likely to lead to overdosing and unintended pharmacological effects. Limitations of our approach need to be acknowledged. First, we derived the evidence from case-series studies and it was not possible to obtain quantitative measures of associations between the B12 dose and the clinical and biomarker responses. Second, the methods used to estimate B12 absorption (based on radioactive B12 as Schilling test or fecal B12) have inherent limitations. However, the studies discussed in the present paper have shown rather consistent results on the amount of B12 absorbed after oral intake. Third, our pragmatic approach can be used for decision making, but the certainty in the evidence is low and the maximal cut-offs suggested in this review might not apply to all individuals. Dose–response studies to define optimal daily B12 intake in people who cannot absorb vitamin B12 might not be doable. Therefore, we consider that future research is unlikely to change our conclusions.

## Conclusion and Recommendations

We reviewed data on pharmacokinetics (the study of how the body interacts with B12, e.g. absorption) and pharmacodynamics (the effects of vitamin B12 on the body) of vitamin B12 within a wide intake range to define the maximal daily intake of B12 from foods, food supplements, and FSMPs. We suggest that food supplements should not contain more than 20 µg. If they contain 50 µg, the product should not be used for longer than 4–6 months. Intakes up to 20 µg/d from food supplements appear to be of low concern regarding interference with metabolism or correction of clinical symptoms, while 50 µg/d might correct abnormal blood count and serum vitamin B12 in some deficient people. The amount of vitamin B12 added to food supplements should not exceed 100 µg at any occasion.

**Fig. 3:**
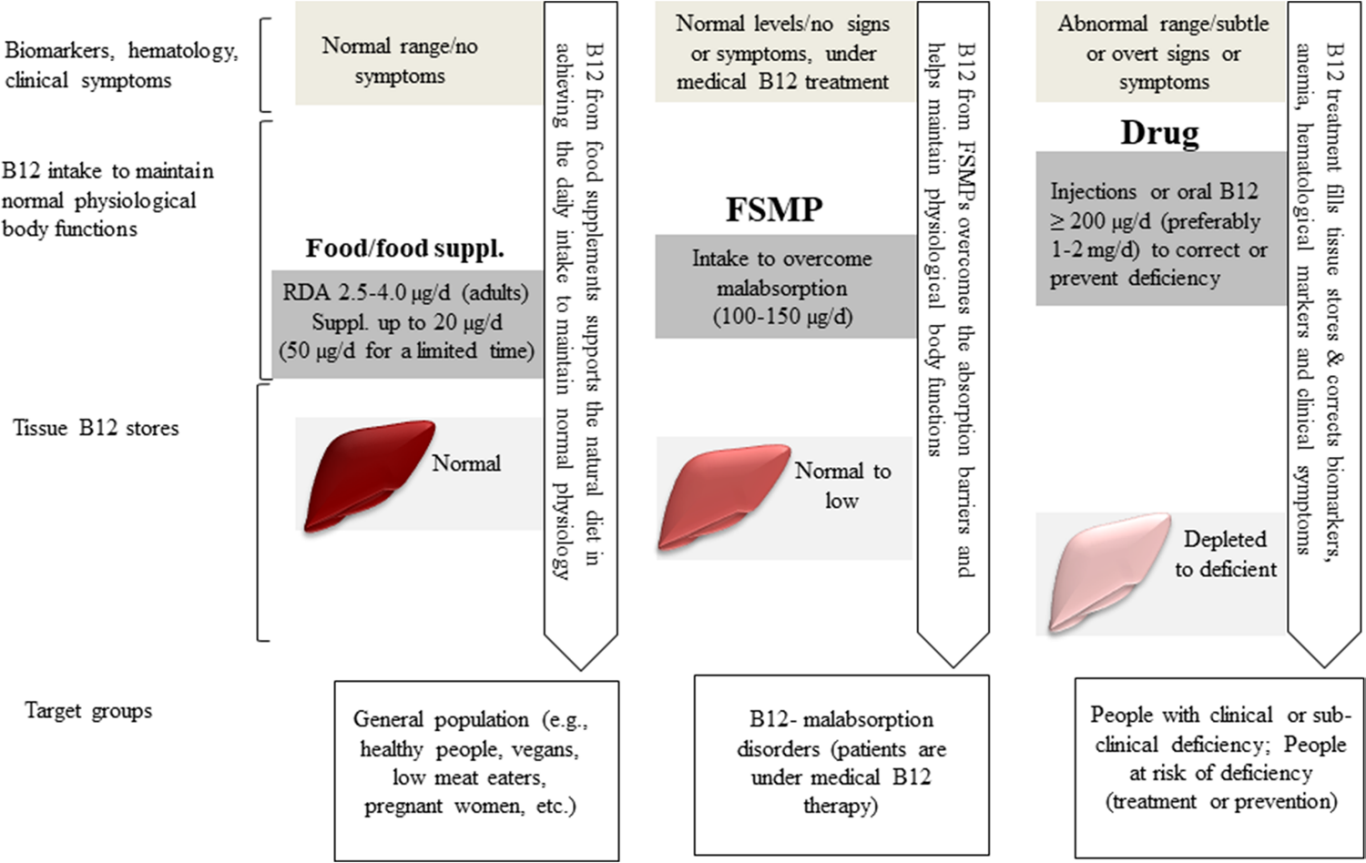
Foods, food supplements, foods for special medical purposes (FSMP), and drugs underlie different definitions and regulations. They do not overlap and their definitions are mutually exclusive. For example, a given oral dose of B12 cannot be used as FSMP and a drug in the same time. Oral vitamin B12 products providing ≥ 200 µg per day are very likely to show pharmacological effects and should not be used in food supplements. There is a consistent dose–response relationship between oral B12 dose and corrections of hematological, metabolic and clinical dysfunctions caused by vitamin B12 deficiency. Food supplements should not provide more than 20 µg B12 per day (50 µg for no longer than 4–6 months)

The FSMP product group may provide more than 50 µg but less than 200 µg vitamin B12 (as a total daily dose), assuming that people who cannot absorb vitamin B12 would achieve roughly 1–4 µg/d of the vitamin reaching the circulation independent of IF. A dose between 100 and 150 µg/d is likely to maintain normal plasma B12 levels in the long term in patients with B12 deficiency due to malabsorption, but may not cause persistent pharmacological effects in this group of patients (Fig. 3). Vitamin B12 intakes ≥ 200 µg/d show pharmacological effects and should not be used in food supplements or FSMPs. This review provides a framework for decision makers to set a maximal amount of B12 in foods.

## Data Availability

No datasets were generated or analysed during the current study.

## Abbreviations

BfR: German Federal Institute for Risk Assessment
EFSA: European Food and Safety Authority
FDA: Food and drug administration
FSMP: Food for special medical purposes
IF: Intrinsic factor
NAM: United States National Academy of Medicine
UL: Tolerable Upper Intake Level
